# The Emotional Lockdown: How Social Distancing and Mask Wearing Influence Mood and Emotion Recognition in Adolescents and Adults

**DOI:** 10.3389/fpsyg.2022.878002

**Published:** 2022-06-10

**Authors:** Louisa Kulke, Theresia Langer, Christian Valuch

**Affiliations:** ^1^Neurocognitive Developmental Psychology, Friedrich-Alexander-Universität Erlangen-Nürnberg, Erlangen, Germany; ^2^Department of Cognition, Emotion, and Methods in Psychology, Faculty of Psychology, University of Vienna, Vienna, Austria

**Keywords:** emotion perception, mood, COVID-19 pandemic, virtual interactions, social contacts

## Abstract

During the COVID-19 pandemic, government-mandated protection measures such as contact restrictions and mask wearing significantly affected social interactions. In the current preregistered studies we hypothesized that such measures could influence self-reported mood in adults and in adolescents between 12 and 13 years of age, who are in a critical phase of social development. We found that mood was positively related to face-to-face but not to virtual interactions in adults and that virtual interactions were associated with negative mood in adolescents. This suggests that contact restrictions leading to a decrease in face-to-face compared to virtual interactions may be related to negative mood. To understand if prolonged exposure to people wearing masks during the pandemic might be related to increased sensitivity for subtle visual cues to others’ emotions from the eye region of the face, we also presented both age groups with the same standardized emotion recognition test. We found slightly better performance in emotion recognition from the eyes in our student sample tested during the pandemic relative to a comparable sample tested prior to the pandemic although these differences were restricted to female participants. Adolescents were also better at classifying emotions from the eyes in the current study than in a pre-pandemic sample, with no gender effects occurring in this age group. In conclusion, while social distancing might have detrimental effects on self-reported mood, the ability to recognize others’ emotions from subtle visual cues around the eye region remained comparable or might have even improved during the COVID-19 pandemic.

## Introduction

In 2020, the COVID-19 pandemic hit the world, leading to sudden societal changes. To contain the virus, people in Germany, as in other parts of the world, were asked to avoid face-to-face contacts with other people and to wear masks (Bundesregierung, 2021). Both of these changes can have effects on social interactions. Humans are inherently social beings (Dunbar, 1998, 2003) and social interaction may even have had evolutionary advantages, in line with the social intelligence hypothesis (Tomasello, 2014). Social contacts can be a protective factor against psychological disorders such as depression (Peirce et al., 2000) and a lack of social contacts has been related to psychological distress (Cacioppo et al., 2014). Increases in psychological disorders and related symptoms have been observed during the pandemic (Rajkumar, 2020; Sønderskov et al., 2020; Vindegaard and Benros, 2020; Xiong et al., 2020; Segre et al., 2021), which may at least partially be related to lack of social contacts among other factors. The lack of *natural social interactions* during the pandemic might be problematic for all age groups but perhaps particularly for adolescents who are still in a vulnerable phase for social development (Segre et al., 2021). Adolescents are particularly sensitive to peer judgment (Somerville, 2013; Somerville et al., 2013; Jones et al., 2014). Furthermore, perspective taking develops as a social skill at this age (van den Bos et al., 2011). In vulnerable phases of development, receiving appropriate social input plays a crucial role in human interactions (Dunbar, 1998, 2003) and may provide evolutionary advantages (Tomasello, 2014). The government-mandated rules during the pandemic might thus, to some degree, prevent healthy social development in adolescence. As adolescents are in a particularly sensitive phase of social development, they may be even more vulnerable to negative effects of the withdrawal from social interactions than adults. A systematic review by Loades et al. (2020) confirms that there is an association between loneliness and mental health problems in previously healthy children and adolescents with the strongest association being found for depression.

Even though people were not allowed to meet face-to-face during the pandemic, they could still meet virtually. Thus, online meetings have often served as a substitute for face-to-face meetings. However, which effects this pandemic-related shift of social interactions into the realm of online meetings might have on personal mood as well as on perceptions of the mood and emotional state of others has not yet been studied systematically. One possibility is that as long as the interaction partners share their video image, at least the access to important emotional facial cues of the other persons might be better in such contexts compared to face-to-face interactions while wearing face masks. It should, however, be noted, that subtle emotional cues and the temporal immediacy of facial expressions in response to an interaction partner may get lost in virtual compared to face to face interactions. Overall, it is currently not well understood how such virtual interactions compare with face-to-face interactions. Previous research showed that at least face-to-face contact could serve as a protective factor against depression, whereas no such effects are so far evident for mere telephone or email contact (Teo et al., 2015). During the pandemic virtual meetings using videocalls increased rapidly, perhaps enabling more natural visual and auditory interactions. Live videocalls activate the social brain (Redcay et al., 2010) and can induce positive affect and physical reactions (Hietanen et al., 2020), suggesting that such interactions are similar to face-to-face social interactions. Accordingly, a recent study suggests that both remote and face-to-face interactions were negatively related to depressive symptoms (Lee et al., 2020), contrasting findings regarding null effects of email and phone contact. Of note, the findings of these previous studies were based on somewhat older age groups and thus might not necessarily generalize to the adolescent and younger adult samples that we tested in the present study. In summary, there are mixed findings in the literature regarding differences between the quality of face-to-face and virtual interactions but studies about neural and emotional responses to face-to-face and video-based communication showed similarities between these interactions.

*Mask wearing*, apart from being an effective measure for reducing virus transmission, also changes the character of social interactions. During social interactions, it is particularly relevant that people understand and react to others’ beliefs and emotions, a skill known as Theory of Mind (ToM; Premack and Woodruff, 1978; Wimmer and Perner, 1983). Emotions are recognized from other people’s body postures, and particularly from their faces (Bänziger et al., 2009). However, important facial cues that are normally available during face-to-face communication were suddenly obscured, since government-mandated wearing of masks led to covering of a large proportion of the face around the mouth and nose (Bundesregierung, 2021). Since large parts of the face are obscured by the face mask, visual cues to the other persons’ intentions or inner mental state that rely on the lower part of the face are inaccessible in such interactions. Indeed, research suggests that perception of emotional expressions is more difficult when parts of the face are covered (Fischer et al., 2012; Kret and De Gelder, 2012). Initial studies during the pandemic also suggest that emotion recognition from faces is impaired when faces were covered by a mask (Carbon, 2020; Ruba and Pollak, 2020; Fitousi et al., 2021), and people need to increasingly rely on cues from the eyes (Barrick et al., 2020; Trainin and Yeshurun, 2021). Humans tend to quickly adapt to environmental change and frequent exposure to masked faces could be related to an improvement in emotion recognition from the eye region of faces. After preregistration of the current study, Trainin and Yeshurun (2021) reported that participants that gained 1 month of experience in interacting with others while wearing masks, significantly improved their emotion recognition skills in a standardized test. Furthermore, gaze cueing effects were significantly increased during the lockdown (Dalmaso et al., 2021), suggesting that the reaction to visual cues in other peoples’ eyes changed during the pandemic. In principle, people could build up considerable experience in interactions with partially masked strangers, but also colleagues and friends due to the pandemic, which might ultimately allow them to improve their recognition of subtle emotion cues from the eyes, perhaps depending on the extent of this exposure.

In our current study, we therefore aimed to investigate the relationship between different types of social interactions during the pandemic (face-to-face vs. virtual, with and without face masks) and the self-reported mood of participants in different age groups (adults and adolescents). We assumed that adolescents might be particularly vulnerable to the drastic changes in social interactions in response to the pandemic. For both age groups, we hypothesized that a lower amount of time spent with other people is associated with negative mood. We further hypothesized that virtual interactions could replace face-to-face interactions. Hence, we did not expect mood to be affected by whether the time with other people was spent in a live or virtual context and that any associations between interaction duration and negative mood should be rather comparable between face-to-face interactions and video-based communication.

In addition to measuring the relationship between social interactions and mood, we were also interested in whether the experience of interacting with others while wearing masks might be positively related to sensitivity for subtle emotional cues that remain visible from the eye region of faces. We hypothesized that emotion recognition from the eyes is better in the sample tested during the COVID-19 pandemic than in a sample tested before the pandemic and that the self-reported time spent with people wearing masks is positively related to emotion recognition from the eyes. Psychological and neuroscientific studies suggest that adolescence is a period of heightened social sensitivity (Somerville, 2013; Somerville et al., 2013; Jones et al., 2014) and significant improvements in social skills occur during adolescence (Redcay and Warnell, 2018). Therefore, this age group may be particularly influenced by the changes in social interactions due to the new rules of mask wearing in social situations. As there is a female advantage in facial emotion perception across the lifespan (Baron-Cohen et al., 1997; Olderbak et al., 2019), we also planned to explore gender differences in the ability to recognize emotions from the eyes.

## Materials and Methods

### Participants

For study 1 on adults, the study’s design, hypotheses and analysis plan were preregistered with the Open Science framework^1^. As preregistered, participants were recruited for 2 weeks (end of February to beginning of March 2021), leading to a sample of *N* = 115 German-speaking adult participants who volunteered to take part in the online questionnaire after informed consent was given. One participant was excluded due to incorrectly entered self-reports of social interaction durations and six participants did not pass the control items after the Reading the Mind in the Eyes Test (RMET), leading to a final adult sample of 108 participants [75 female, age: 18.30--69.82 years, *M* = 30.64, *SD* = 15.77, 95% CI (27.64, 33.65)]. Participants could win one of two 15€ vouchers^2^ and receive course credit for their participation. During the survey of the adult study, Germany was in lockdown. Schools, kindergartens, and universities were closed and students only had online classes. Most workers had to work in home office. Stores, restaurants and other leisure facilities were closed. Contact restrictions only allowed people to meet with one other household with a maximum of five people in total. Furthermore, in regions from which the participants were mostly recruited, there were time restrictions on when you were allowed to leave your house (not between 9/10 pm and 5 am). Mask wearing and distance rules applied in all public buildings, transport, and places (Bundesgesundheitsministerium, 2021; Bundesregierung, 2021). At the end of February the 7-days-incidence was Germany-wide at around 64 cases per 100,000 inhabitants (Robert-Koch-Institut, 2021).

For study 2 on adolescents, design, hypotheses and analysis plan were also preregistered with the Open Science framework (see text footnote 1). *N* = 91 German-speaking adolescent participants between 12.00 and 13.99 years [*M* = 12.99, *SD* = 0.59, 95% CI (12.85, 13.12)] were recruited *via* the recruitment platform kinderschaffenwissen.de, Facebook groups, emails to the parent councils of local schools and *via* local contacts (from March 2021 until June 2021). They volunteered to take part in the online questionnaire after informed consent was given by the participants and by their guardians. 29 adolescents did not fill out the whole questionnaire and therefore had to be excluded. Furthermore, seven participants who were younger than the required age range and five participants who were older were excluded from our analyses. Furthermore, three participants who did not pass two control items after the RMET were excluded, so that the final adolescent sample consisted of 76 participants (49 females and 27 males). Participants could win one of two 15€ vouchers (see text footnote 2) for their participation. During the survey of the adolescent study, some of the restrictions that had been in place during the adult survey were loosened again. Schools started to reopen for normal classes in the middle of March, with students and teachers having to wear masks in the beginning. At the end of March masks were only mandatory during the breaks and when one left their seat. Due to the rising number of COVID-19 cases and the quarantine rules for contact persons, schools frequently switched between distance and face-to-face teaching. Stores, restaurants, and other leisure facilities were allowed to open with restrictions only at a low regional incidence. People were still obligated to limit their number of social contacts, with changing restrictions depending on the number of cases in the area, and the vaccination status. The rules were different depending on the federal state and became stricter again as soon as an area was declared a hot spot with a 7-day-incidence above 100. Mask wearing and distance rules applied in all public buildings, transport, and places (Bundesgesundheitsministerium, 2021; Bundesregierung, 2021). Throughout Germany, the 7-days-incidence per 100,000 inhabitants reached values of around 130 (end of March), 158 (end of April), and around 40 (end of May; Robert-Koch-Institut, 2021).

### Study Design

In an observational between-participants design, the average self-reported time spent with people with and without masks for a typical week was measured for face-to-face and virtual situations. The circumstances (mask wearing, time in virtual and face to face interactions with or without masks) differed between participants. Further dependent variables (details below) were the ability to recognize emotion from the eyes measured through the score on the RMET – Adult Version (Baron-Cohen et al., 1997) and the current negative mood measured through the score on the Aktuelle Stimmungsskala (ASTS; Dalbert, 2002). Sociodemographic variables were included as control variables as well as for exploratory analyses.

The current adolescent sample was additionally compared with an adolescent sample that was tested before the pandemic [*n* = 36, *M*_age_ = 12.73, *SD*_age_ = 0.57, 95% CI (12.54, 12.92), 15 females and 21 males] (Valuch and Kulke, 2020). Furthermore, a subsample including only university students between 18 and 25 years of age [*n* = 65, *M* = 21.26 years, *SD* = 2.32, 95% CI (20.68, 21.83), 50 females and 15 males] was compared with a recent student sample tested before the pandemic [Open Data by Valuch and Kulke (2020), *n* = 55, *M*_age_ = 22.15, *SD*_age_ = 2.17, 95% CI (21.56, 22.73), 35 females and 20 males]. In addition, the full adult sample of the present study was compared with the mean of the neurotypical sample reported by Baron-Cohen et al. (2001; *n* = 122, *M*_age_ = 46.5, *SD*_age_ = 16.9).

### Stimuli

#### Reading the Mind in the Eyes Test

The RMET is usually associated with ToM ability and is often used for the screening of autism spectrum disease; it is also suitable for use with adolescent participants (Baron-Cohen et al., 1997). It consists of a practice item with solution followed by 36 further items. Each item shows a black and white photograph of the eye region of a person’s face presented with four words describing emotional expressions. The participants need to choose the expression they think describes best the mental state of the person in the photograph. The total score is calculated from the sum of the items correctly answered. We used the original paper-pencil-test as an online version that was conducted as closely to the original version as possible. Several studies only found poor internal consistency and heterogeneity of the RMET (Olderbak et al., 2015), but the complete German version of the RMET had a satisfactory test-retest reliability (*r* = 0.68; Pfaltz et al., 2013). Research on convergent validity consistently identified the RMET as being related to emotion perception (Pfaltz et al., 2013; Olderbak et al., 2015) and maybe even measuring emotion recognition rather than ToM ability (Olderbak et al., 2019). Since the aim of this study was to investigate the effects of mask wearing on emotion perception from the eyes, the RMET seemed to be a suitable measuring tool.

#### Measuring Different Types and Durations of Interaction

Participants were asked to estimate how much time they spend on each day of a typical week interacting in the following four different categories: face-to-face interactions with people who were wearing masks; face-to-face interactions with people who were not wearing masks; digital interactions with people wearing masks and digital interactions with people not wearing masks. Examples for digital and face-to-face contacts were given with digital contacts being defined as conversations or interactions *via* computer, smartphone, tablet, or other technical devices (e.g., online meetings or video chats with your friends) and face-to-face contacts being defined as contacts with people who were in the same place as the participant (e.g., while shopping, talking to the family or a roommate). Participants reported hours and minutes separately for each category for every day. For the analyses, estimations were averaged across the week for each category.

#### Mood Questionnaire

The “ASTS” is a German short version of the Profile of Mood States (originally by McNair et al., 1971). It was optimized and shortened through a factor analysis and records the state part of subjective wellbeing (Dalbert, 2002). It consists of 19 items, rated on a scale from 7 (very strong) to 1 (not at all). The items form five subscales [Sorrow (S), Hopelessness (H), Fatigue (F), Rage (R), and Positive Mood (P)]. The sum of the reversed Positive Mood subscale and the other subscales except for Rage constitute the global score for current negative mood (ASTS_Score). Internal consistency measures suggest a sufficient reliability of the subscales and there are indications of differential and construct validity (Dalbert, 2002).

### Procedure

Questionnaires were implemented using SoSci Survey (Leiner, 2019) and could be filled in on computers and smartphones. Adults provided informed consent before starting the study. For adolescents, first, guardians provided an informed consent for their children to participate, and afterward children themselves provided an informed consent and entered their demographic data. Guardians were asked not to get involved in the study during testing. After providing informed consent and demographic information, participants completed the RMET, followed by two control items, which were similar to RMET items but had an easy and unambiguously correct answer to check task compliance. Participants then estimated the time they spend with people in face-to-face and virtual situations with and without masks for each day of a typical week and filled in the ASTS mood questionnaire.

#### Data Preparation

Data retrieved from SoSci Survey were prepared for analysis in R (version 4.0.2). *RMET score* and *ASTS score* were computed according to the manuals. For two participants, a typo was corrected^3^. It did not make a difference if the persons who made typos were excluded or if the mistakes were corrected, therefore we included the participants and corrected their mistakes. The average interaction time (in hours) for a typical day of the different interaction categories was computed. The following indices were computed: The proportion of time spent with people wearing masks was computed separately for face-to-face/online/and both situations. For the analysis of mood hypotheses, we additionally computed a separate variable coding the proportion of face-to-face in relation to all interactions for each participant. Bayes factors (BF_10_) comparing the full model with the model without the respective effect of interest and effect sizes were computed using the BayesFactor package (Morey and Rouder, 2015).

#### Analysis Plan

Statistical analyses were conducted using R/RStudio (version 4.0.2; R Core Team, 2021). Independent samples *t*-tests using the *t*.test function of the stats package (R Core Team, 2021) were used to compare the RMET Score between the pre-pandemic and the pandemic samples. For both studies an ANOVA was conducted to investigate the effects of gender and sample on the RMET Score using the ez package (Lawrence, 2016). For group comparisons using *t*-tests or ANOVAs normality assumptions were checked graphically through histograms, Q-Q-plots and through Shapiro–Wilk-Test and variance homogeneity was checked through Levene’s test.

For both studies, several linear models were computed using the *lm* function of the *lmtest* package (Zeileis and Hothorn, 2002), with one to predict RMET Score from the proportional time spent with people wearing masks in face-to-face situations, another one to predict RMET Score from the proportional time spent with people wearing masks in online situations and a third one was to predict RMET Score from the overall proportional time spent with people wearing masks. In both studies a linear model was computed to predict negative mood measured through the ASTS Score from the overall time spent interacting with people and the variable coding the proportion of face-to-face interaction among the overall interaction time. Assumptions regarding normality of the residuals, linearity of the correlation and homoscedasticity were checked graphically. However, for all linear models, robust standard errors were computed using the coeftest function from the lmtest package (Zeileis and Hothorn, 2002) to fix potential heteroscedasticity.

## Results of Study 1: Adults

### Descriptive Statistics

Table 1 shows descriptive statistics for socio-demographics, RMET score, ASTS score, and the different interaction categories.

**TABLE 1:** Means, standard deviations, confidence intervals and correlations for age, housemates, RMET score, ASTS score and the different interaction time variables (adults, *n* = 108).

				Correlations							
				
	*M*	*SD*	95% CI	1.	2.	3.	4.	5.	6.	7.	8.
1. Age (years)	30.64	15.77	27.63, 33.65	1.00							
2. Housemates^a^	2.88	1.32	2.63, 3.13	−0.27**	1.00						
3. RMET score	25.81	3.51	25.14, 26.48	−0.55***	0.06	1.00					
4. ASTS score	52.06	16.64	49.80, 56.15	−0.22*	0.01	0.20*	1.00				
5. Time face-to-face with mask (hours)^b^	1.15	1.53	0.86, 1.45	0.11	–0.00	–0.05	–0.03	1.00			
6. Time face-to-face without mask (hours)^b^	6.81	5.08	5.84, 7.77	0.02	0.20*	–0.02	−0.27**	0.05	1.00		
7. Time virtual with mask (hours)^b^	0.03	0.18	-0.01, 0.06	0.03	–0.18	–0.18	–0.16	0.10	–0.12	1.00	
8. Time virtual without mask (hours)^b^	1.41	1.1	1.20, 1.62	–0.18	0.01	0.04	0.26**	−0.19*	–0.18	0.07	1.00

*p < 0.05, **p < 0.01, and ***p < 0.001.

*^a^Number of people living together with the study participants including themselves.*

*^b^Time estimations for the different categories averaged over a typical week.*

### Relationship Between Interactions and Mood in the Pandemic Sample

**FIGURE 1:**
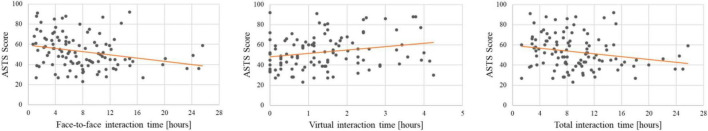
Linear regression analyses for the current negative mood predicted from face-to-face, virtual, and total interaction time in adults (*n* = 108).

A linear model was computed to predict negative mood measured through the ASTS Score from the overall time spent interacting with people and the variable coding the proportion of face-to-face interaction among the overall interaction time. Proportion of face-to-face interaction among all interactions was a significant predictor for the current negative mood (*b* = –33.55, β = –0.37, SE = 9.77, *t* = –3.43, *p* = 0.001), whereas the overall time spent with other people did not have a significant effect (*b* = –0.12, β = –0.04, SE = 0.29, *t* = –0.41, *p* = 0.680), model: *F* (2, 105) = 8.94, *p* < 0.001, adjusted *R*^2^ = 12.93%, BF = 96.872. An exploratory analysis with the overall time spent in face-to-face interactions and the overall time spent in virtual interactions as predictors, *F* (2, 105) = 5.71, *p* = 0.004, adjusted *R*^2^ = 8.09%, BF = 7.396, revealed that mood was better if more time was spent with people face-to-face (*b* = –0.68, β = –0.22, SE = 0.28, *t* = –2.44, *p* = 0.016), while virtual interactions had no significant relation to mood (*b* = 2.60, β = 0.18, SE = 1.58, *t* = 1.64, *p* = 0.104), see Figure 1.

### Emotion Recognition From the Eyes – Comparison Between Pre-pandemic and Pandemic Sample

An independent *t*-test to compare the RMET Score of the student subsample of the adult study with the Open Data of adults collected by Valuch and Kulke (2020) was conducted. It revealed a significantly higher mean of the RMET Score in our student subsample tested during the pandemic than in the sample tested before the pandemic [Pre-pandemic sample: *M*_1_ = 25.82, *SD*_1_ = 3.41, 95% CI_1_ (24.90, 26.74), Pandemic Sample: *M*_2_ = 26.92, *SD*_2_ = 3.15, 95% CI_2_ (26.14, 27.70), *t* (118) = 1.85, *p* = 0.068, note that this two-sided *p*-value is significant compared to the preregistered *p*_*crit*_ = 0.10, 95% CI (–0.08, 2.29), *d* = 0.34, BF = 0.897]. The one sample *t*-test to compare our full sample with the mean values of typical adults tested by Baron-Cohen et al. (2001) was not significant [*M* = 26.2, current sample: *M* = 25.81, *SD* = 3.51, *t* (107) = –1.14, *p* = 0.256, 95%CI (25.15, 26.48), *d* = 0.11, BF = 0.201].

An exploratory linear model, *F* (4, 115) = 4.96, *p* = 0.001, adjusted *R*^2^ = 11.7%, BF = 12.040, to control for further possible influences in the comparison between our student sample and the sample tested by Valuch and Kulke (2020) included gender (*b* = –1.67, SE = 0.67, *t* = –2.50, *p* = 0.014), age (*b* = –0.12, SE = 0.14, *t* = –0.86, *p* = 0.393), psychology student status (*b* = 1.13, SE = 0.76, *t* = 1.50, *p* = 0.137), and the sample (before or during the pandemic; *b* = –0.42, SE = 0.65, *t* = –0.65, *p* = 0.516) as predictors. In this model, only gender was a significant predictor for the RMET Score.

### Relationship Between Interactions and Emotion Recognition From the Eyes in the Pandemic Sample

Linear models to predict the ability to recognize emotions from the eyes (RMET Score) from the proportion of time spent with people wearing masks in face-to-face interactions, *F* (1, 106) = 0.72, *p* = 0.399, *R*^2^ = 0.67%, BF = 0.281, and from the proportion of time spent with people wearing masks among all interactions, *F* (1, 106) = 1.23, *p* = 0.270, *R*^2^ = 1.15%, BF = 0.353, were not significant. The linear model predicting emotion recognition from the eyes from the proportion of time spent with people wearing masks in digital interactions was significant, *F* (1, 106) = 4.03, *p* = 0.047, *R*^2^ = 3.67%, BF = 1.217, but contrary to our hypothesis. The effect furthermore seems unreliable due to the small variation in the proportion of time with most people not spending any time in virtual conversations with people wearing masks.

### Planned Exploratory Analyses–Comparison Between Pre-pandemic and Pandemic Sample

A two-way ANOVA was conducted to predict the RMET score from gender (female vs. male) and sample [full current study (2) vs. study by Valuch and Kulke, 2020 (1)], showing a female superiority in the RMET [*M*_female1_ = 26.20, *SD*_female1_ = 3.27, 95% CI (25.08, 27.32), *M*_female2_ = 26.49, *SD*_female2_ = 3.37, 95% CI (25.72, 27.27), *M*_male1_ = 25.15, *SD*_male1_ = 3.62, 95% CI (23.46, 26.84), *M*_male2_ = 24.27, *SD*_male2_ = 3.36, 95% CI (23.08, 25.46), *F* (1, 159) = 10.14, *p* = 0.002, *f* = 0.25, BF = 17.352]. The main effect of sample, *F* (1, 159) = 0.04, *p* = 0.847, *f* = 0.02, BF = 0.178, and the interaction effect between gender and sample, *F* (1, 159) = 0.98, *p* = 0.323, *f* = 0.08, BF = 0.389, were both not significant.

Multiple linear regression analyses were conducted to explore whether time spent with others (face-to-face and/or digital) relates differently to the different subscales of the ASTS mood questionnaire (Sorrow, Hopelessness, Fatigue, Positive Mood, and Rage). Of the five subscales the multiple regression analyses for *Hopelessness, F* (2, 105) = 5.37, *p* = 0.006, adjusted *R*^2^ = 7.55%, *B*_10_ = 5.592, *Sorrow*, *F* (2, 105) = 7.37, *p* = 0.001, adjusted *R*^2^ = 10.64%, *B*_10_ = 28.08, and *Fatigue*, *F* (2, 105) = 4.30, *p* = 0.016, adjusted *R*^2^ = 5.81%, *B*_10_ = 2.332, were significantly related to *overall* time spent with others. Only the time spent in *face-to-face interactions* was a significant predictor for the current feeling of hopelessness (*b* = –0.17, SE = 0.059, β = –0.23, *t* = –2.81, *p* = 0.006), while the time spent in *digital interactions* was not significant (*b* = 0.50, SE = 0.39, β = 0.15, *t* = 1.27, *p* = 0.208). Similarly, the overall face-to-face interaction time was negatively related to the current sorrow (*b* = –0.25, SE = 0.67, β = –0.30, *t* = –3.82, *p* < 0.001), while the digital interaction time was not related to sorrow (*b* = 0.48, SE = 0.39, β = 0.12, *t* = 1.24, *p* = 0.218). For fatigue only the digital interaction time was a significant predictor (*b* = 1.37, SE = 0.50, β = 0.27, *t* = 2.76, *p* = 0.007), while the face-to-face interaction time was not (*b* = –0.00, SE = 0.12, β = –0.00, *t* = –0.04, *p* = 0.971). The overall model for *Positive Mood* was not significant, *F* (2, 105) = 2.53, *p* = 0.085, adjusted *R*^2^ = 2.77%, *B*_10_ = 0.531, however, the time spend in face-to-face interactions was positively related to the current positive mood (*b* = 0.26, SE = 0.12, β = 0.20, *t* = 2.10, *p* = 0.038).

### Exploratory Analyses – Comparison Between Pre-pandemic and Pandemic Sample

Because of the gender differences in the student sample and the sample by Valuch and Kulke (2020) and the role of gender in the process of emotion recognition from the eyes, we divided the student sample and the sample of Valuch and Kulke (2020) in a female and a male subsample. The independent sample *t*-test for the male subsample was not significant [*M*_1_ = 25.15, *SD*_1_ = 3.62, 95% CI (23.46, 26.84), *M*_2_ = 24.47, *SD*_2_ = 2.88, 95% CI (22.87, 26.06), *t* (33) = –0.60, *p* = 0.551, 95% CI (–2.99, 1.63), *d* = 0.21, BF = 0.377], but the one for the females was significant [*M*_1_ = 26.20, *SD*_1_ = 3.27, 95% CI: (25.08, 27.32), *M*_2_ = 27.66, *SD*_2_ = 2.86, 95% CI (26.85, 28.47), *t* (83) = 2.18, *p* = 0.032, 95% CI (0.13, 2.79), *d* = 0.48, BF = 1.777].

## Results Study 2: Adolescents

### Descriptive Statistics

Results did not differ depending on how outliers were handled or whether the individuals who did not answer the control item correctly were excluded. Therefore, as preregistered, the participants who did not pass both control items after the RMET were excluded but no further adjustments to the outliers were made.

Table 2 shows means, standard deviations, 95%-CIs and correlations between socio-demographics, RMET score, ASTS score, and the time spent in different situations (digital or face-to-face) with and without wearing a mask.

**TABLE 2:** Means, Standard deviations, confidence intervals and correlations for age, housemates, RMET score, ASTS score and the different interaction time variables for the adolescent sample (*n* = 76).

				Correlations							
				
	*M*	*SD*	95% CI	1.	2.	3.	4.	5.	6.	7.	8.
1. Age (years)	12.99	0.59	12.85, 13.12	1.00							
2. Housemates^a^	4.07	0.98	3.84, 4.29	0.11	1.00						
3. RMET score	23.58	3.42	22.80, 24.36	0.17	–0.01	1.00					
4. ASTS score	53.16	17.64	49.13, 57.19	–0.10	0.00	–0.09	1.00				
5. Time face-to-face with mask (hours)^b^	2.14	1.87	1.71, 2.56	–0.07	–0.08	–0.04	–0.19	1.00			
6. Time face-to-face without mask (hours)^b^	8.01	5.76	6.70, 9.33	0.16	–0.11	–0.08	0.17	–0.20	1.00		
7. Time digital with mask (hours)^b^	0.15	0.48	0.04, 0.26	0.23*	0.23*	0.09	–.011	0.00	–0.15	1.00	
8. Time digital without mask (hours)^b^	2.36	2.13	1.87, 2.84	0.01	–0.04	–0.03	0.22*	−0.62***	0.12	–0.11	1.00

*p < 0.05 and ***p < 0.001.

*^a^Number of people living together with the study participants including themselves.*

*^b^Time estimations for the different categories averaged over a typical week.*

### Relationship Between Interactions and Mood in the Pandemic Sample

The same multiple linear regression analysis as for the adults was conducted for the adolescents to predict *ASTS score*, which represents the current negative mood from the overall time spent interacting with people and the variable coding the proportion of this time that was spend in face-to-face interactions. The multiple linear regression analysis was not significant, *F* (2, 73) = 1.76, *p* = 0.180, adjusted *R*^2^ = 1.98%, *B*_10_ = 0.347. Neither the proportion of face-to-face interaction among all interactions (*b* = –11.27, β = –0.12, SE = 14.54, *t* = –0.77, *p* = 0.441), nor the overall time spent with other people (*b* = 0.65, β = 0.22, SE = 0.38, *t* = 1.72, *p* = 0.090) was a significant predictor for the current negative mood.

### Planned Exploratory Analyses of Mood in the Pandemic Sample

Multiple linear regression analyses with the overall time spent in face-to-face interactions and the overall time spent in virtual interactions as variables were conducted to explore whether time spent with others (face-to-face and/or digital) relates differently to the different subscales of the ASTS mood questionnaire. Of the five subscales (Sorrow, Hopelessness, Fatigue, Positive Mood, and Rage) the multiple regression analyses for Hopelessness, *F* (2, 73) = 4.79, *p* = 0.011, adjusted *R*^2^ = 9.19%, *B*_10_ = 0.491, and Fatigue, *F* (2, 73) = 3.17, *p* = 0.048, adjusted *R*^2^ = 5.48%, *B*_10_ = 1.077, were significant. Only the time spent in digital interactions was a significant predictor for the current feeling of hopelessness (*b* = 0.73, SE = 0.34, β = 0.33, *t* = 2.13, *p* = 0.036), while the time spent in face-to-face interactions was not significant (*b* = 0.12, SE = 0.10, β = 0.15, *t* = 1.26, *p* = 0.212). Similarly, the overall digital interaction time was positively related to the current fatigue (*p* = 0.021), but when taking the robust standard errors into account to fix heteroscedasticity, the time spent in digital interactions was no longer a significant predictor (*b* = 0.74, SE = 0.43, β = 0.27, *t* = 1.73, *p* = 0.088). The face-to-face time was not related to fatigue (*b* = 0.14, SE = 0.43, β = 0.13, *t* = 1.12, *p* = 0.266).

### Emotion Recognition From the Eyes – Comparison Between Pre-pandemic and Pandemic Sample

An independent sample *t*-test revealed a significantly higher mean of the RMET Score in the adolescent sample tested during the COVID-19 pandemic than the sample tested before the pandemic [*M*_Valuch&Kulke_ = 21.89, *SD*_Valuch&Kulke_ = 4.71, 95% CI_Valuch&Kulke_ (20.29, 23.48), *M*_pandemic_ = 23.58, *SD*_pandemic_ = 3.42, 95% CI_pandemic_ (22.80, 24.36), *t* (110) = 2.15, *p* = 0.033, 95% CI (0.14, 3.24), *d* = 0.44, *B*_10_ = 1.632], supporting our hypothesis. An additional analysis compared 200 randomly selected sub-samples of the current datasets with the dataset by Valuch and Kulke (2020). 106 of the 200 random subsamples (each *n* = 36) were significantly better at emotion recognition from the eyes than the sample tested before the pandemic and all comparisons had a positive direction, indicating, that all 200 subsamples had at least the same or a higher mean than the sample tested before the pandemic by Valuch and Kulke (2020).

### Relationship Between Interactions and Emotion Recognition From the Eyes in the Pandemic Sample

Three simple linear regression analyses were conducted to investigate if the self-reported time spent with people wearing masks is positively related to emotion recognition from the eyes. For each analysis, *RMET score* was the dependent variable. The independent variables for the three regression analyses were: the proportion of time spent with people wearing masks in face-to-face situations, the proportion of time spent with people wearing masks in digital interactions and the proportion of time spent with people wearing masks overall. The results of the linear regression analyses can be seen in Table 3. Contrary to our hypothesis, none of the three linear models was significant. Therefore, in the current sample the exposure to people wearing masks was not associated with better emotion recognition from the eyes.

**TABLE 3:** Results of the linear models predicting the RMET Score from the proportion of time spent in different interactions while wearing masks in adolescents.

	RMET score			

Predictors	*F* ^a^	*p*	*R^2^ (%)*	*B_10_*
Time face-to-face with mask	0.15	0.703	0.20	0.253
Time digital with mask	0.00	0.975	0.01	0.238
Overall time with mask	0.15	0.701	0.20	0.253

*^a^df = 74 for all models.*

### Gender Differences in Emotion Recognition From the Eyes – Comparison Between Pre-pandemic and Pandemic Sample

An ANOVA was conducted to investigate whether female participants are better at emotion recognition from the eyes than males in both samples. Neither the main effect of gender, *F* (1,108) = 0.59, *p* = 0.445, *f* = 0.07, *BF*_10_ = 0.385, nor the interaction effect with sample, *F* (1, 108) = 0.13, *p* = 0.715, *f* = 0.04, *BF*_10_ = 0.312, were significant. However, the analysis confirmed a higher RMET score during the pandemic compared to the pre-pandemic sample [*M*_female1_ = 22.00, *SD*_female1_ = 4.74, 95% CI (19.28, 24.62), *M*_female2_ = 23.86, *SD*_female2_ = 3.38, 95% CI (22.89, 24.83), *M*_male1_ = 21.81, *SD*_male1_ = 4.81, 95% CI (19.62, 24.00), *M*_male2_ = 23.07, *SD*_male2_ = 3.50, 95% CI (21.69, 24.46), *F* (1, 108) = 3.71, *p* = 0.057, *f* = 0.19, *B*_10_ = 1.632; significant based on the preregistered *p* = 0.10 cut-off for one-sided tests]. This suggests that the sample tested during the pandemic was better in emotion recognition from the eyes than the sample tested before the pandemic by Valuch and Kulke (2020).

To explore potential effects of age differences between samples, an exploratory linear model to control for possible influences in the comparison between our sample and the sample tested by Valuch and Kulke (2020) included *age* (*b* = 0.97, SE = 0.66, *t* = 1.48, *p* = 0.142) and *sample* (*b* = –31.14, SE = 16.94, *t* = –1.84, *p* = 0.069) as predictors and also included a possible interaction between the two predictors (*age × sample*: *b* = 2.33, SE = 1.31, *t* = 1.79, *p* = 0.077; overall model: *F* (3, 108) = 5.26, *p* = 0.002, adjusted *R*^2^ = 10.32%, *B*_10_ = 10.939). In this model, only sample was a significant predictor for the RMET Score, while the interaction effect only reached marginal significance. However, to further control for a potential age influence on the RMET score, age-related matched pairs for the RMET score in the two studies [pre-pandemic sample (*M*_1_ = 21.89, *SD*_1_ = 4.71, 95% CI_1_ (20.29, 23.48)) vs. pandemic subsample *(M_2_* = 22.89, *SD*_2_ = 3.43, 95% CI_2_ (21.73, 24.05), *n* = 36)] were compared using another independent sample *t*-test. The *t*-test was not significant, *t* (70) = –1.03, *p* = 0.307, 95% CI (–2.94, 0.94), *d* = 0.24, *B*_10_ = 0.383.

### Exploratory Analyses in the Pandemic Sample

Since mood effects differed between the adolescent sample and the previously tested adult sample, differences between the adolescents and adults in the time spent in digital interactions were investigated using an independent sample *t*-test. Results indicated that adolescents spend significantly more time in virtual interactions than adults do [*M*_adults_ = 1.44, *SD*_adults_ = 1.13, 95% CI_adults_ (1.22, 1.65), *M*_adolescents_ = 2.51, *SD*_adolescents_ = 2.13, 95% CI_adolesencts_ (2.02, 2.99), *t* (104) = –3.99, *p* < 0.001, 95% CI (–1.60, –0.54), *d* = 0.66, *B*_10_ = 978.265].

### Exploratory Analysis: Mood Differences Between the Age Groups in the Pandemic Sample

To investigate whether the influence of social contacts on the current mood differs between adolescents and adults, a linear model was conducted with the overall time spent interacting with people, age and the interaction between both variables as independent variables and mood measured through the ASTS Scale as the dependent variable. The model did not reach significance [*F* (3, 180) = 1.68, *p* = 0.172, adjusted *R*^2^ = 1.11%, BF = 0.107; factors: *interaction time*:, *age: b* = –0.02, β = –0.16, SE = 0.17, *t* = –0.13, *p* = 0.900, *interaction effect*: *b* = –0.01, β = –0.07, SE = 0.01, *t* = –1.17, *p* = 0.246], indicating that the overall time (face-to-face and digital) spent with people was not a significant predictor for mood regardless of the age.

### Exploratory Analysis: Differences in Reading the Mind in the Eyes Test Scores Between Age Groups

Since adolescence is a phase of heightened social sensitivity, younger people could possible be better at adapting to the pandemic situation. Possible differences in the improvement of the ability to recognize emotions from the eyes between adults and adolescents due to the mask wearing were examined, using a two-way ANOVA to predict the RMET score from age-group (adult students vs. adolescents) and sample [pandemic (2) vs. pre-pandemic by Valuch and Kulke, 2020 (1)] [*M*_adolescents1_ = 21.89, *SD*_adolescents1_ = 4.71, *n*_adolescents1_ = 36, *M*_adolescents2_ = 23.58, *SD*_adolescents2_ = 3.42, 95% CI (), *n*_adolescents2_ = 76, *M*_adults1_ = 25.82, *SD*_adults1_ = 3.41, *n*_adults1_ = 55, *M*_adults2_ = 26.92, *SD*_adults2_ = 3.15, *n*_adults2_ = 65]. The ANOVA showed a significant main effect of age-group, *F* (1, 228) = 56.56, *p* < 0.001, *f* = 0.50, and of the sample on the RMET, *F* (1, 228) = 7.94, *p* = 0.005, *f* = 0.19. The interaction effect between age-group and sample, *F* (1, 228) = 0.36, *p* = 0.549, *f* = 0.04, was not significant. This indicates that adults were overall better at emotion recognition from the eyes than younger adolescents and that both age-groups were significantly better at this skill when tested during the pandemic than before.

## Discussion

The current study investigated the relationship between the extent of social interactions in face-to-face and virtual settings and self-reported mood in the context of government-mandated restrictions during the COVID-19 pandemic. In addition, because wearing of face masks obscures important emotional cues from faces, we were also interested if pandemic-caused exposure to masked faces might be positively related to sensitivity for subtle emotional cues from the eye region that remains visible. We collected the same measures in adults (study 1) and adolescents (study 2), with the latter being an age group that is particularly sensitive to social development. Before we discuss the results in more depth, we briefly summarize the main findings.

In our *adult* sample tested during the COVID-19 pandemic, mood was better if more time was spent with people in face-to-face interactions, whereas virtual interactions had no such positive relation to mood. Emotion recognition from the eyes was marginally better in the current sample compared to a recent pre-pandemic sample but there was no relation between the time spent with people wearing masks and emotion recognition from the eyes. The previously reported female superiority in emotion recognition from the eyes was replicated. Moreover, the improvement in emotion recognition from the eyes seemed to be stronger for the female students.

In our *adolescent sample*, there was no relation between time spent with other people and overall mood; however, the time spent in virtual interactions was positively related to hopelessness and fatigue. Adolescents’ emotion recognition from the eyes was also significantly better in the current study tested during the pandemic, than in a sample tested shortly before the pandemic. However, there was no direct relation between the self-reported time spent with people wearing masks and emotion recognition from the eyes. Gender did not play a role for emotion recognition.

### Relationship Between Interactions and Mood

We originally hypothesized that the time one spends with other people is positively related to mood, independent of whether one meets face-to-face or virtually. This hypothesis was not confirmed by our data, as in adults only the time spent in face-to-face situations was positively related to mood. This finding is in line with previous studies which suggested that face-to-face interactions have benefits for psychological well-being (Cacioppo et al., 2014). It is also unlikely that this finding could be explained by the fact that people were generally in a better mood when they interacted more with housemates, as the correlation between the mood score and the amount of housemates was not significant (see Table 1).

In contrast, adolescents showed no relation between social contacts and *overall* mood. One possibility could be that adolescents adapt better to the novel situation and therefore suffer less from reduced social contacts. It could also be that they are better prepared to pursue social interactions virtually compared to adults, which seems to be supported by our exploratory comparison of time spent in virtual interactions. Crucially, however, adolescents’ time spent in virtual interactions was also related to the subscales of the mood questionnaire “hopelessness” and “fatigue,” suggesting that virtual interactions do not have a positive effect on mood, but rather a negative one on certain aspects of it. As virtual interactions did not have a comparable positive effect as face-to-face interactions but, in contrast, tended to be negatively related to mood, merely interacting with others virtually cannot simply replace face-to-face interactions. Thus, with respect to their effects on mood, the video-based interactions regarded in the current study might not necessarily be superior to phone or email contacts that were investigated in previous research (Teo et al., 2015). It should be noted, that the exploratory comparison of adolescents’ and adults’ interaction effects on mood was not significant; however, this interaction was not originally preregistered and therefore the study was not planned for this comparison and may underestimate effects.

While the current study was the first to collect data relating time spent in virtual and face-to-face interactions to mood and emotion recognition, it has some important limitations. For example, we did not collect further information on whether face-to-face and virtual interactions were with friends, family, work associates or other people. Due to the home office recommendation in Germany, many virtual meetings may be work-related or school-related and as university classes were moved online, virtual meetings may also be study-related in the student population, as teaching was moved online. An alternative explanation is therefore, that only meetings with friends have a positive effect on mood but not meetings in a work-related or study-related context. Since the first lockdown and contact restrictions video-based communication tools have enjoyed rising popularity (Statista, 2021), making it easier and relevant to investigate differences between video-based and face-to-face communication. This cross-sectional study reported some interesting preliminary findings; however, future research should longitudinally collect information about the time spent in virtual and face-to-face interactions and not only rely on self-reports.

### Emotion Recognition From the Eyes

As people spend more time interacting with others wearing masks, we hypothesized that they may develop improved emotion recognition from the eyes; however, this was only partially observed in the current set of studies. It is also important to note that the current studies were purely observational and not experimental. As all people were simultaneously affected by the pandemic, and a controlled manipulation of mask exposure was not possible. A slight improvement of emotion recognition from the eyes is in line with the observations of a study published after our preregistrations that found improvements with exposure to people wearing masks (Trainin and Yeshurun, 2021). In general, the current studies did not find a relation between mask exposure and RMET score in adults or adolescents and no interaction effects with age groups. However, the variance within our sample may have been too small as all participants were exposed to others wearing masks to a similar degree. Furthermore, both samples included different participants, tested at different points in time. Historic or demographic factors may therefore have contributed to the observed findings.

In particular, in adolescents, exploratory analyses suggest that emotion recognition effects may also be related to slight age differences between the samples rather than to effects of the pandemic. In adults, another demographic factor that may have contributed to the emotion recognition effects was gender. In addition to replicating the female superiority in emotion recognition from the eyes (Kirkland et al., 2013; Olderbak et al., 2019) in adults we observed that the differences between the samples before and during the pandemic were highest in female students. The general female superiority may be helpful for quicker or better adaptation to the current situation, leading to improvements in emotion recognition from the eyes due to the mask-wearing only for female students. As the separate analyses for different genders were exploratory, they should be interpreted with caution and more studies are necessary to appropriately investigate the possible relationship between gender and adaptation to the pandemic situation. No such female superiority effect was observed in the adolescent sample. This is interesting, as such effects may develop with age. They may therefore either be culturally shaped or related to hormonal changes occurring during puberty. A study investigating adolescents up to 15 years of age showed gender effects (Olderbak et al., 2019); however, the participants in this study were slightly older than in the current study, so that a significant developmental change may occur during puberty. Furthermore, gender effects may have been overestimated in previous studies, or may only exist for presentation of the view of the whole face, but not for the eye region itself, as studies and meta analyses using the RMET only found small or even no gender effects on emotion recognition from the eyes (Kirkland et al., 2013; Pfaltz et al., 2013). As adolescents are in a sensitive phase, they may generally adapt to the new situation more quickly, leading to a lack of gender interactions.

### Limitations of Our Observational Design

The participants tested in the current pandemic sample were compared with a different sample tested before the pandemic. Although this between-participant comparison has the advantage of avoiding familiarity effects in the Reading Mind in the Eyes that could occur as a result of prior experience to the test materials, it also has the disadvantage that differences between the samples may have led to the observed effects. Since we observed people at two different time points with differing circumstances, and we did not experimentally manipulate any of the factors, we cannot draw inferences about the causality of the effects. To give one example, people who have a negative mood might have less face-to-face interactions, while people with more positive mood might have more contacts with others, which could produce a pattern of results similar to the one that we observed. Also, the sample tested in our current study was on average younger than samples tested in previous research on mood; therefore the groups should be compared with caution.

## Conclusion

In summary, our cross-sectional observational study investigated relations of the sudden societal changes during the pandemic, replacement of face-to-face with virtual interactions and wearing of masks on mood and emotion recognition from eyes in adults and adolescents. Mood showed a tendency to be positively related to face-to-face but negatively to virtual social interactions, suggesting that the positive psychological effect of social interactions cannot simply be replaced by virtual interactions of any kind. The ability to recognize emotion from the eyes was better during the pandemic than before in both age groups, but in adults this improvement only seemed to appear for females who were also generally better at emotion recognition from the eyes. In summary, the COVID-19 related contact restrictions may have affected adolescents who are in a particularly sensitive social development period, and also adults, but further research is required to confirm these effects.

## Data Availability Statement

Primary data are available through the Open Science Framework (https://osf.io/7g4ds/).

## Ethics Statement

Ethical review and approval was not required for the study on human participants in accordance with the local legislation and institutional requirements. Written informed consent to participate in this study was provided by the participants or the participants’ legal guardian/next of kin.

## Author Contributions

LK, TL, and CV contributed to conception and design of the study. TL performed the statistical analysis, supervised by LK and CV. LK wrote the first draft of the manuscript. All authors contributed to manuscript revision, read, and approved the submitted version.

## Conflict of Interest

The authors declare that the research was conducted in the absence of any commercial or financial relationships that could be construed as a potential conflict of interest.

## Publisher’s Note

All claims expressed in this article are solely those of the authors and do not necessarily represent those of their affiliated organizations, or those of the publisher, the editors and the reviewers. Any product that may be evaluated in this article, or claim that may be made by its manufacturer, is not guaranteed or endorsed by the publisher.

## Footnotes

1
http://osf.io/7g4ds

2
Wunschgutschein.de

3 One participant accidently filled in the estimations for *hours* and *minutes* together in one field. Another participant’s smartphone autocorrected a zero to *OK*, which we corrected.
